# LncRNA GAS5 relates to Th17 cells and serves as a potential biomarker for sepsis inflammation, organ dysfunctions and mortality risk

**DOI:** 10.1002/jcla.24309

**Published:** 2022-03-24

**Authors:** Weizhen Zhang, Bingqing Chen, Wei Chen

**Affiliations:** ^1^ Intensive Care Unit Longhua Hospital Affiliated to Shanghai University of Traditional Chinese Medicine Shanghai China; ^2^ Internal Medicine Department Yueyang Hospital of Integrated Traditional Chinese and Western Medicine Shanghai University of Traditional Chinese Medicine Shanghai China

**Keywords:** APACHE II, LncRNA GAS5, mortality risk, SOFA and its subscales, Th1 and Th17 cells

## Abstract

**Background:**

Long noncoding RNA GAS5 (lnc‐GAS5) is able to regulate macrophage M1 polarization and Th17 cell differentiation, also engaged in sepsis‐induced inflammation and organ injury. This study aimed to further evaluate its linkage with Th1 cells and Th17 cells, as well as its clinical value in sepsis management.

**Methods:**

About 101 sepsis patients were enrolled followed by peripheral blood mononuclear cell (PBMC) and serum samples collection. PBMC lnc‐GAS5 was detected by RT‐qPCR; Th1 cells and Th17 cells in PBMC CD4^+^ T cells were detected by flow cytometry; serum IFN‐γ and IL‐17A were detected by ELISA. Besides, PBMC lnc‐GAS5 was also detected in 50 health controls (HCs).

**Results:**

Lnc‐GAS5 was reduced in sepsis patients than in HCs (*p* < 0.001), which also well‐distinguished sepsis patients from HCs with AUC 0.860. Lnc‐GAS5 did not relate to Th1 cells (*p* = 0.059) or IFN‐γ (*p* = 0.192); while negatively linked with Th17 cells (*p* = 0.002) and IL‐17A (*p* = 0.019) in sepsis patients. Interestingly, lnc‐GAS5 negatively correlated with SOFA score (*p* = 0.001), SOFA‐Respiratory system score (*p* = 0.001), SOFA‐Coagulation score (*p* = 0.015), and SOFA‐Renal system score (*p* = 0.026), but not SOFA‐Liver score (*p* = 0.080), SOFA‐Cardiovascular system score (*p* = 0.207) or SOFA‐Nervous system score (*p* = 0.182) in sepsis patients. Furthermore, lnc‐GAS5 was negatively related to CRP (*p* = 0.002) and APACHE II score (*p* = 0.004) in sepsis patients. Finally, lnc‐GAS5 was decreased in dead sepsis patients compared to survivors (*p* = 0.007), which also distinguished sepsis deaths from survivors with AUC 0.713.

**Conclusion:**

Lnc‐GAS5 relates to Th17 cells and serves as a potential biomarker for sepsis severity and mortality risk.

## Graphical abstract

One hundred‐one sepsis patients were enrolled for detecting lnc‐GAS5, Th1 cells, Th17 cells, IFN‐γ and IL‐17A. Besides, 50 health controls (HCs) were also enrolled for detecting lnc‐GAS5. Lnc‐GAS5 was reduced in sepsis patients than in HCs. Lnc‐GAS5 negatively linked with Th17 cells and IL‐17A; negatively correlated with SOFA score, SOFA‐Respiratory system score, SOFA‐Coagulation score and SOFA‐Renal system score; negatively related to CRP and APACHE II score in sepsis patients. Lnc‐GAS5 decreased in died sepsis patients compared to survivors.

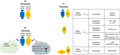

## 1 INTRODUCTION

Sepsis is featured by life‐threatening organ dysfunction with an extremely dysregulated host response to infection.^1^ The mortality of sepsis is unacceptably high with 28 days mortality ranging from 8.5% to 31.6%, whose prevention and treatment have been regarded as a global health priority by the World Health Organization (WHO), recently.^2^, ^3^, ^4^, ^5^, ^6^, ^7^, ^8^, ^9^ With a deepened understanding of the pathogenesis of sepsis, the differentiation of T cells, polarization of macrophage, and the inflammation response are thought to be closely involved in the development and progression of sepsis,^10^, ^11^, ^12^ therefore, finding a biomarker that might regulate these aforementioned biological processes during sepsis is helpful for the management of sepsis to further improve its prognosis.

Long noncoding RNA GAS5 (lnc‐GAS5) is firstly reported to act as a regulator of the malignant phenotype of cancer cells.^13^, ^14^ However, with more comprehensive studies being performed, its anti‐inflammation role has been proposed. Recent studies disclose that lnc‐GAS5 inhibits the differentiation of T cells into Th17 cells, macrophage M1 polarization while promotes macrophage M2 polarization, and restrains the inflammation as well as sepsis‐induced organ injury in multi‐complex diseases, which implies that it might be engaged in the pathogenesis of sepsis.^15^, ^16^, ^17^, ^18^, ^19^, ^20^, ^21^ Nevertheless, the clinical involvement of lnc‐GAS5 in sepsis remains unclear. Hence, this study aimed to further evaluate its linkage with Th1 cells and Th17 cells, as well as its clinical value in sepsis management.

## 2 METHODS

### 2.1 Subjects

This study was a prospective cohort study. This study serially enrolled 101 sepsis patients who were treated in our hospital from July 2018 to January 2021. The patients with the following criteria were eligible for the study: (A) confirmed as sepsis according to the sepsis‐3 criteria published in 2016,^22^ (B) more than 18 years old, (C) were hospitalized within 24 h of symptom onset. The exclusion criteria included: (A) had cancer or hematological disease, (B) had a prior history of radiotherapy, chemotherapy or immunotherapy, (C) concomitant with autoimmune disease (these patients might have a dysregulated lnc‐GAS5 expression), (D) women with pregnant or lactating. For comparison, the study also enrolled 50 healthy subjects without clinical evidence of infection as healthy controls during the same period. The enrollment criteria for healthy controls were (A) matched age and gender to sepsis subjects, (B) no history of sepsis, (C) normal biochemical indexes level. Besides, the healthy controls were also ineligible for the study if they met the above exclusion criteria for sepsis patients. The study was approved by the Ethics Committee of Longhua Hospital Affiliated with Shanghai University of Traditional Chinese Medicine. The written informed consents were collected from all subjects or their legal guardians.

### 2.2 Data collection

After enrollment, clinical features were collected, including demographics, comorbidities, disease characteristics, biochemistry indexes, Acute Physiology and Chronic Health Evaluation II (APACHEII) score and Sepsis‐related Organ Failure Assessment (SOFA) score. Besides, all sepsis patients were followed up for 28 days, and the deaths of patients within 28 days were recorded.

### 2.3 Sample collection

For sepsis patients, peripheral blood (PB) samples were collected immediately after admission, then peripheral blood mononuclear cells (PBMCs) and serum were isolated, respectively. In detail, PBMCs were isolated from peripheral blood with a density gradient technique; serum was isolated by centrifugal separation at 1000 g for 20 min. For healthy controls, PB samples were collected immediately after enrollment, then PBMCs were also separated from peripheral blood with a density gradient technique.

### 2.4 Flow cytometry

Within 12h after PBMCs separation in sepsis patients, The CD4 positive (CD4^+^) T cells, Th1 cells and Th17 cells in PBMCs were labeled with specific fluorescent antibody in HumanTh1/Th17 Phenotyping Kit (BD Pharmingen™, BD,), and the labeled cells were counted by flow cytometry analysis. Then, the proportion of Th1 and Th17 cells in CD4^+^ T cells was calculated. The detailed experimentation was strictly in accordance with the manufacturer's instructions.

### 2.5 Enzyme‐linked immunosorbent assay (ELISA)

After serum isolation in sepsis subjects, the level of interferon‐gamma (IFN‐γ) and Interleukin 17A (IL‐17A) were measured by ELISA using Human IFN‐γ Quantikine ELISA Kit and Human IL‐17A Quantikine ELISA Kit (R&D Systems Europe, Ltd.,), respectively. All experiments were carried out based on the direction of instructions.

### 2.6 Reverse transcription quantitative polymerase chain reaction (RT‐qPCR) assay

After PBMC separation in all subjects, lnc‐GAS5 expression was assessed by RT‐qPCR assay. The total RNA isolation and subsequent reverse transcription were conducted by PureZOL RNA isolation reagent (Bio‐Rad, Hercules) and PrimeScript™ RT reagent Kit (Takara, Dalian), respectively. Then, a qPCR assay was carried out by SYBR^®^ Green Realtime PCR Master Mix (Toyobo,). The β‐actin was used as the internal reference with a 2^−ΔΔCt^ method for the calculation of the lnc‐GAS5 expression. The primers were designed according to the previous study.^23^ In detail, the sequence of primers was as follows: lnc‐GAS5, forward primer (5'‐>3’): TGAAGTCCTAAAGAGCAAGCC, reverse primer (5'‐>3’): ACCAGGAGCAGAACCATTAAG; β‐actin, forward primer (5'‐>3’): TCGTGCGTGACATTAAGGAG, reverse primer (5'‐>3’): GTCAGGCAGCTCGTAGCTCT.

### 2.7 Statistics

SPSS (24.0 version, IBM Corp.,) was employed for statistical analysis, and GraphPad Prism (6.01 version, GraphPad Software Inc.,) was applied for graph construction. Differences of variates between groups were compared using Mann–Whitney *U* test. Associations between variates were analyzed using Spearman's rank correlation test, Mann–Whitney *U* test or Kruskal–Wallis H rank sum test. The ability of variables in distinguishing different subjects was estimated using receiver operating characteristic (ROC) curve analysis. Logistic regression analysis was performed to explore the independent factor predicting the sepsis risk. *p* value less than 0.05 was considered statistical significance.

## 3 RESULTS

### 3.1 Clinical features

Among the 101 enrolled sepsis patients, their age was 57.1 ± 11.7 years with 71 (70.3%) males and 30 (29.7%) females (Table 1). In terms of their primary infection site, 33 (32.7%), 31 (30.7%), 18 (17.8%), and 19 (18.8%) patients were diagnosed with abdominal infection, respiratory infection, skin and soft tissue infection, as well as other infections. Regarding the primary organism, 53 (52.5%), 23 (22.8%), 7 (6.9%), and 16 (15.8%) patients were diagnosed as G−, G+, fungus, and other organism‐caused infection, while the rest 17 (16.8%) patients were total culture negative. The APACHE II score was 11.5 ± 5.5, meanwhile, the SOFA score was 4.7 ± 1.9. Other detailed information was listed in Table 1.

**TABLE 1:** Clinical features

Items	Sepsis patients (*N* = 101)
Demographics	
Age (years), mean ± SD	57.1 ± 11.7
Gender, No. (%)	
Female	30 (29.7)
Male	71 (70.3)
BMI (kg/m^2^), mean ± SD	22.8 ± 3.7
Smoke status, No. (%)	
Never	65 (64.3)
Former	23 (22.8)
Current	13 (12.9)
Comorbidities	
History of hypertension, No. (%)	42 (41.6)
History of hyperlipidemia, No. (%)	15 (14.9)
History of diabetes, No. (%)	9 (8.9)
History of CKD, No. (%)	7 (6.9)
History of cardiovascular and cerebrovascular diseases, No. (%)	15 (14.9)
Disease characteristics	
Primary infection site, No. (%)	
Abdominal infection	33 (32.7)
Respiratory infection	31 (30.7)
Skin and soft tissue infection	18 (17.8)
Other infections	19 (18.8)
Primary organism, No. (%)	
G−	53 (52.5)
G+	23 (22.8)
Fungus	7 (6.9)
Others	16 (15.8)
Total culture negative	17 (16.8)
Biochemistry indexes	
Scr (mg/dL), median (IQR)	1.5 (1.1–2.5)
Albumin (g/L), median (IQR)	26.1 (20.8–35.3)
WBC (10^9^/L), median (IQR)	21.3 (14.5–31.3)
CRP (mg/L), median (IQR)	68.7 (35.4–106.8)
Health status scores	
APACHE II score, mean ± SD	11.5 ± 5.5
SOFA score, mean ± SD	4.7 ± 1.9

Abbreviations: APACHE II, Acute Physiology and Chronic Health Evaluation II; BMI, body mass index; CKD, chronic kidney disease; CRP, C‐reactive protein; G‐, gram‐negative bacillus; G+, gram‐positive bacillus; IQR, interquartile range; Scr, serum creatinine; SD, standard deviation; SOFA, Sequential Organ Failure Assessment; WBC, white blood cell.

### 3.2 lnc‐GAS5 expression

**FIGURE 1:**
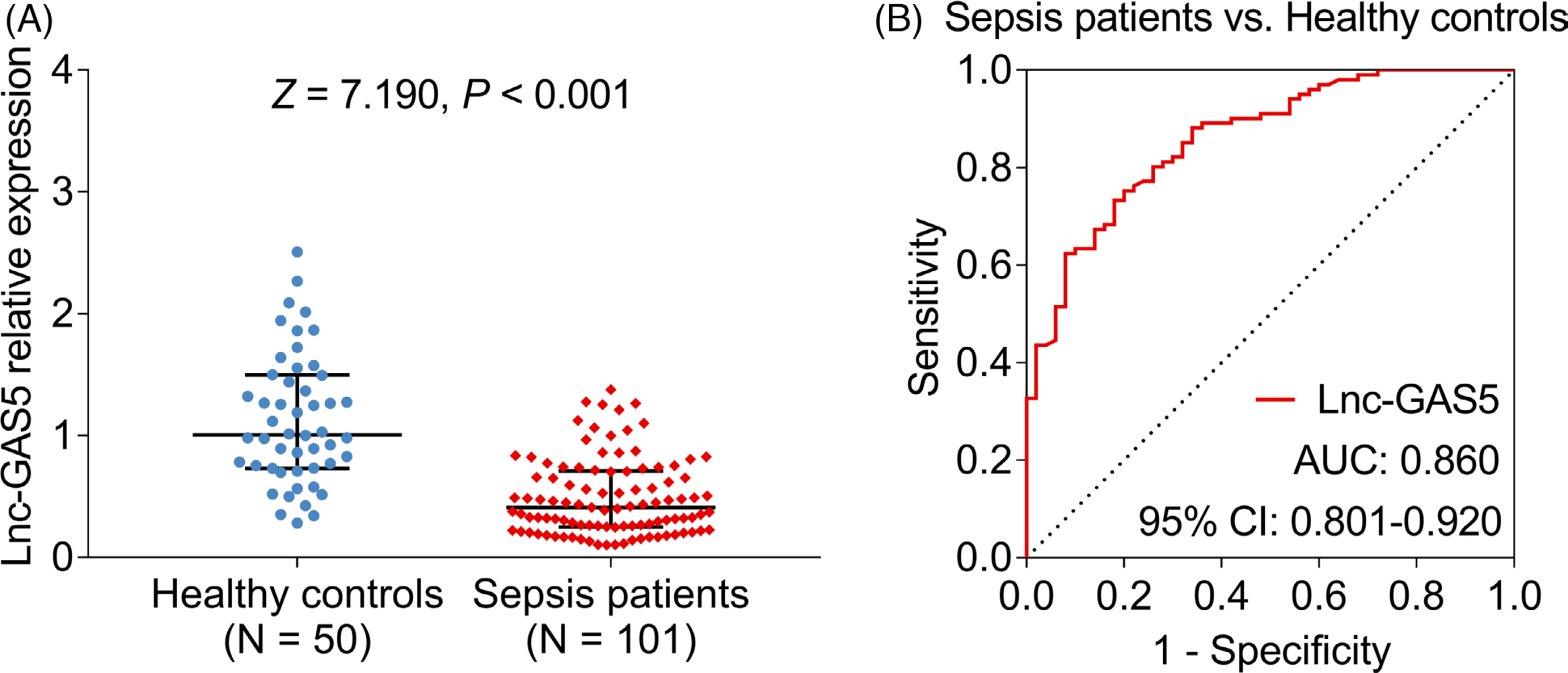
lnc‐GAS5 in distinguishing sepsis patients from healthy controls. Comparison of lnc‐GAS5 expression between sepsis patients and healthy controls (A). The ability of lnc‐GAS5 in differentiating sepsis patients from healthy controls (B)

lnc‐GAS5 expression was decreased in sepsis patients compared with healthy controls (0.410 (interquartile range (IQR): 0.249–0.711) vs. 1.007 (IQR: 0.732–1.497), *p* < 0.001, Figure 1A). The further ROC curve analysis disclosed that lnc‐GAS5 exhibited a good diagnosis value of sepsis with an area under the curve (AUC): 0.860 (95% confidential interval (CI): 0.801–0.920, Figure 1B). Besides, a logistic regression analysis was also performed, which showed that lnc‐GAS5 was an independent factor in predicting the sepsis risk (Table S1).

### 3.3 Correlation of lnc‐GAS5 with the Th cells, organ injuries, and inflammatory status

**FIGURE 2:**
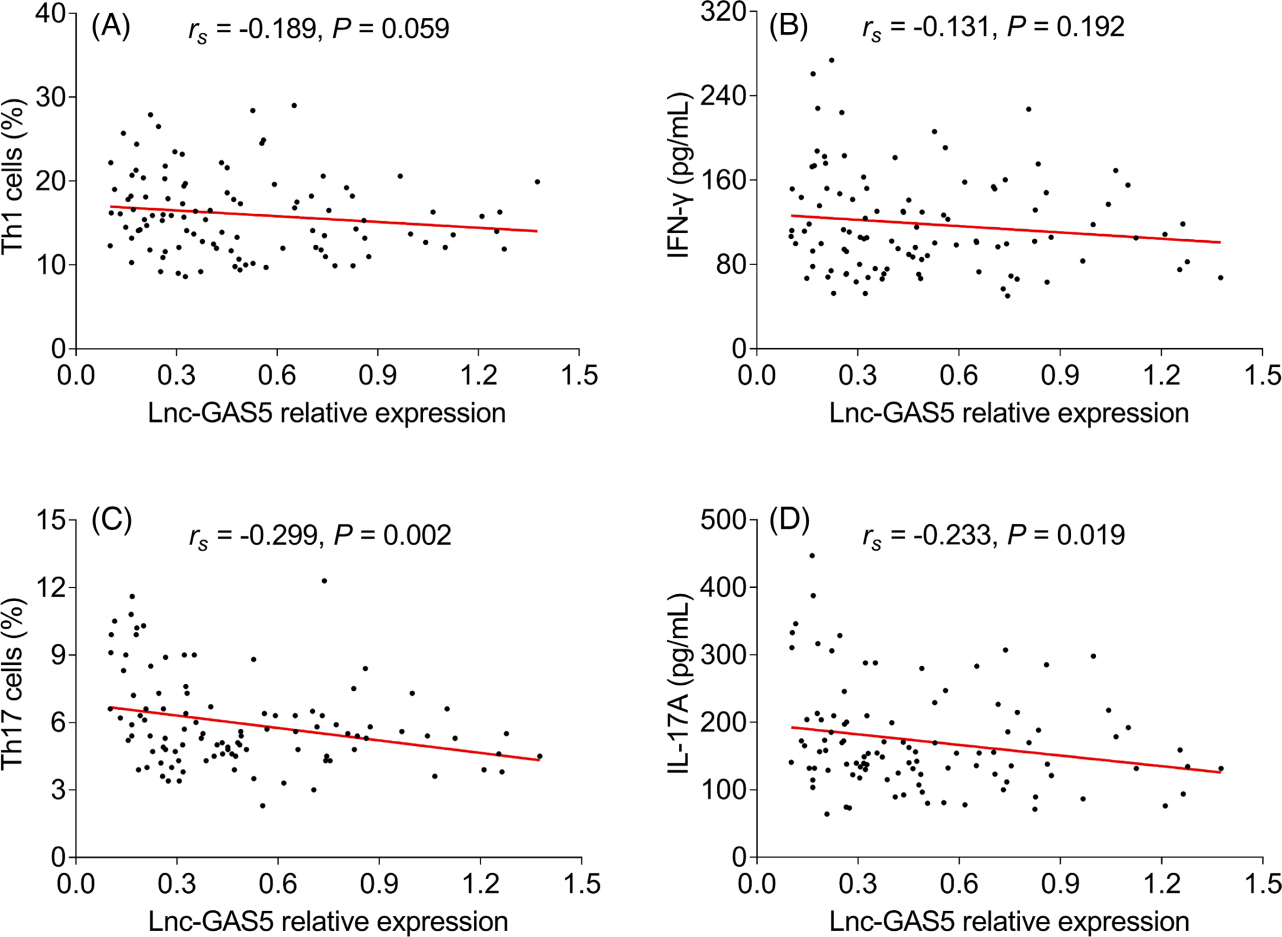
lnc‐GAS5 correlates with Th17 cells and IL‐17A in sepsis patients. Correlation of lnc‐GAS5 expression with Th1 (A), IFN‐γ (B), Th17 cells (C), IL‐17A (D) in sepsis patients

**FIGURE 3:**
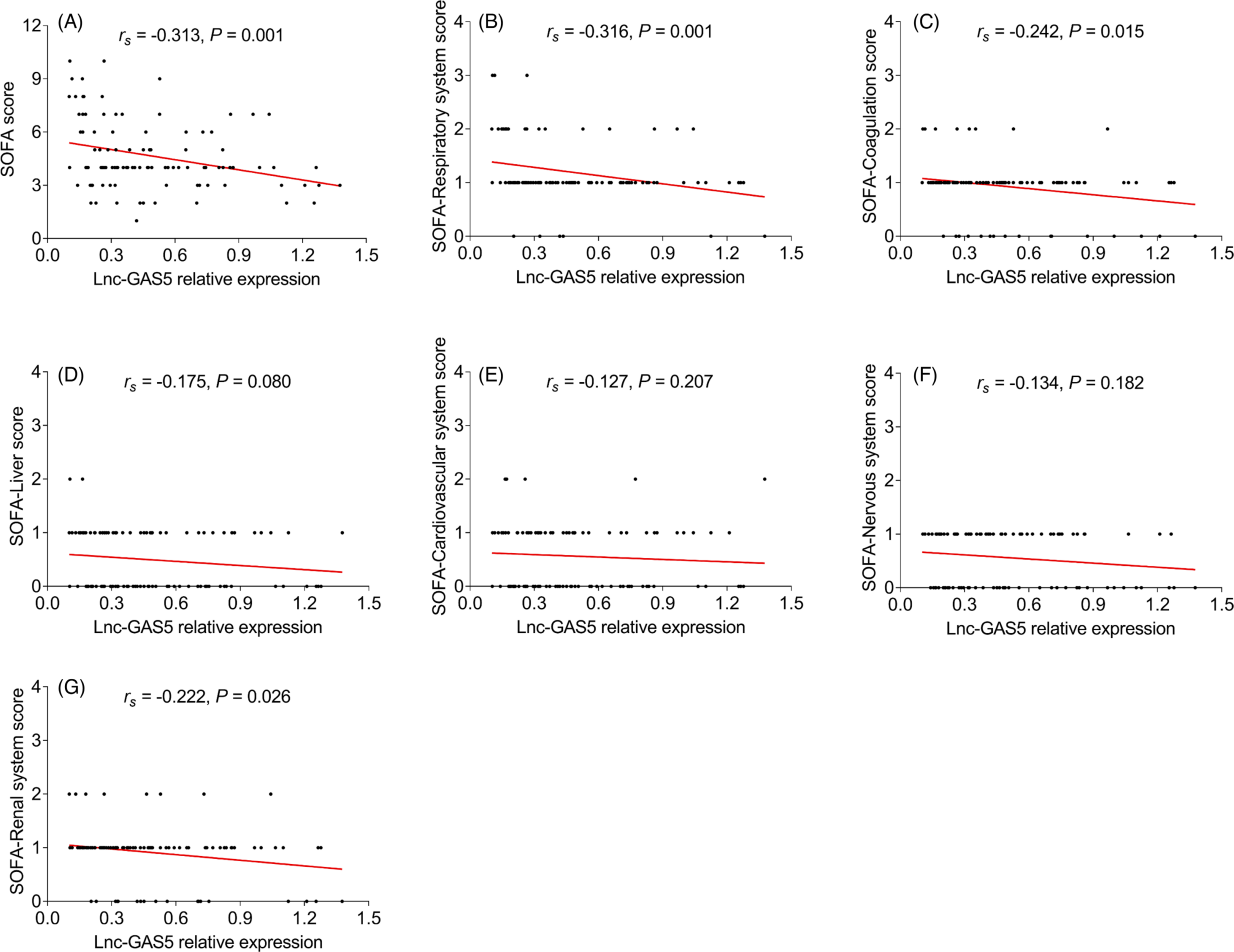
lnc‐GAS5 correlates with SOFA score and some of its subitems. Correlation of lnc‐GAS5 expression with SOFA score (A), SOFA‐Respiratory system score (B), SOFA‐Coagulation score (C), SOFA‐Liver score (D), SOFA‐Cardiovascular system score (E), or SOFA‐Nervous system score (F), and SOFA‐Renal system score (G) in sepsis patients

**FIGURE 4:**
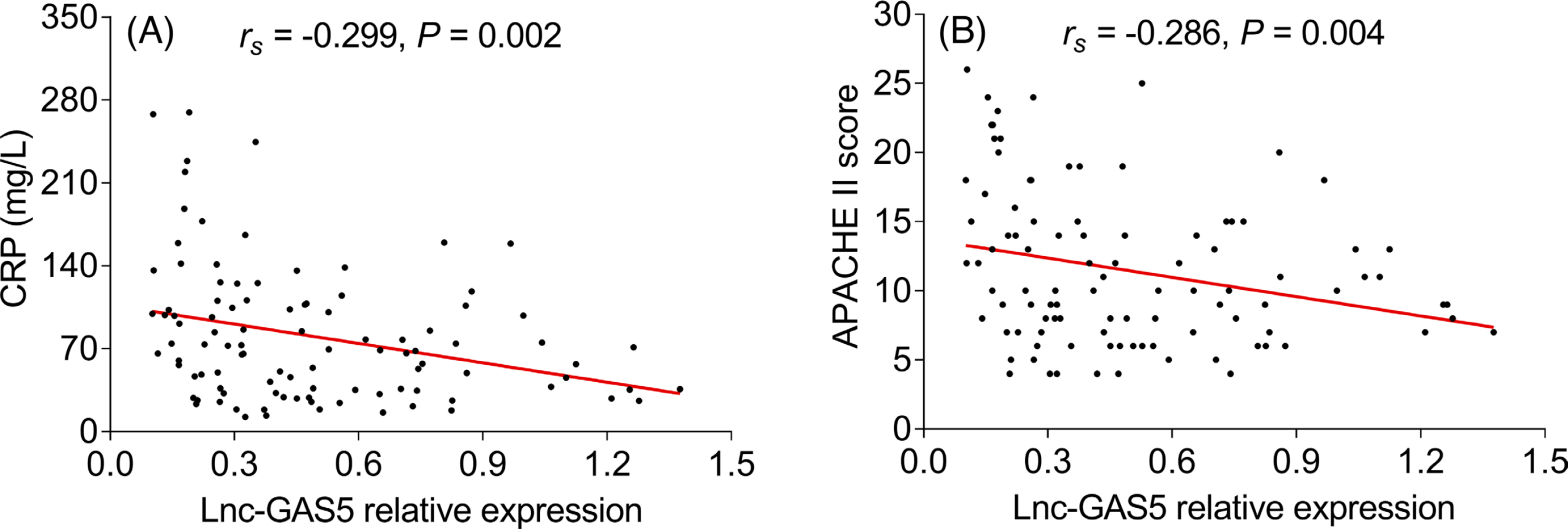
lnc‐GAS5 correlates with CRP and APACHE II score. Correlation of lnc‐GAS5 expression with CRP (A) and APACHE II score (B) in sepsis patients

lnc‐GAS5 was not associated with Th1 cells or IFN‐γ (both *p* > 0.05), while negatively correlated with Th17 cells (*r_s_* = −0.299, *p* = 0.002) and IL‐17A (*r_s_* = −0.233, *p* = 0.019, Figure 2A–D). Apart from that, it was observed that lnc‐GAS5 negatively related to SOFA score (*r_s_* = −0.313, *p* = 0.001), and some of its subitems including SOFA‐Respiratory system score (*r_s_* = −0.316, *p* = 0.001), SOFA‐Coagulation score (*r_s_* = −0.242, *p* = 0.015), and SOFA‐Renal system score (*r_s_* = −0.222, *p* = 0.026); but not correlated with SOFA‐Liver score, SOFA‐Cardiovascular system score, or SOFA‐Nervous system score (all *p* > 0.05, Figure 3A–G). Besides, it was also disclosed that lnc‐GAS5 was negatively associated with C‐reactive protein (CRP) (*r_s_* = −0.299, *p* = 0.002) and APACHE II score (*r_s_* = −0.286, *p* = 0.004, Figure 4A–B).

The correlation of lnc‐GAS5 expression with other clinical features was also of great interest in this study, which indicated that elevated lnc‐GAS5 expression was associated with the fungus negative (*p* = 0.031), while lnc‐GAS5 expression was not correlated with other clinical features (all *p* > 0.05, Table S2).

### 3.4 lnc‐GAS5 in predicting the 28‐day mortality

**FIGURE 5:**
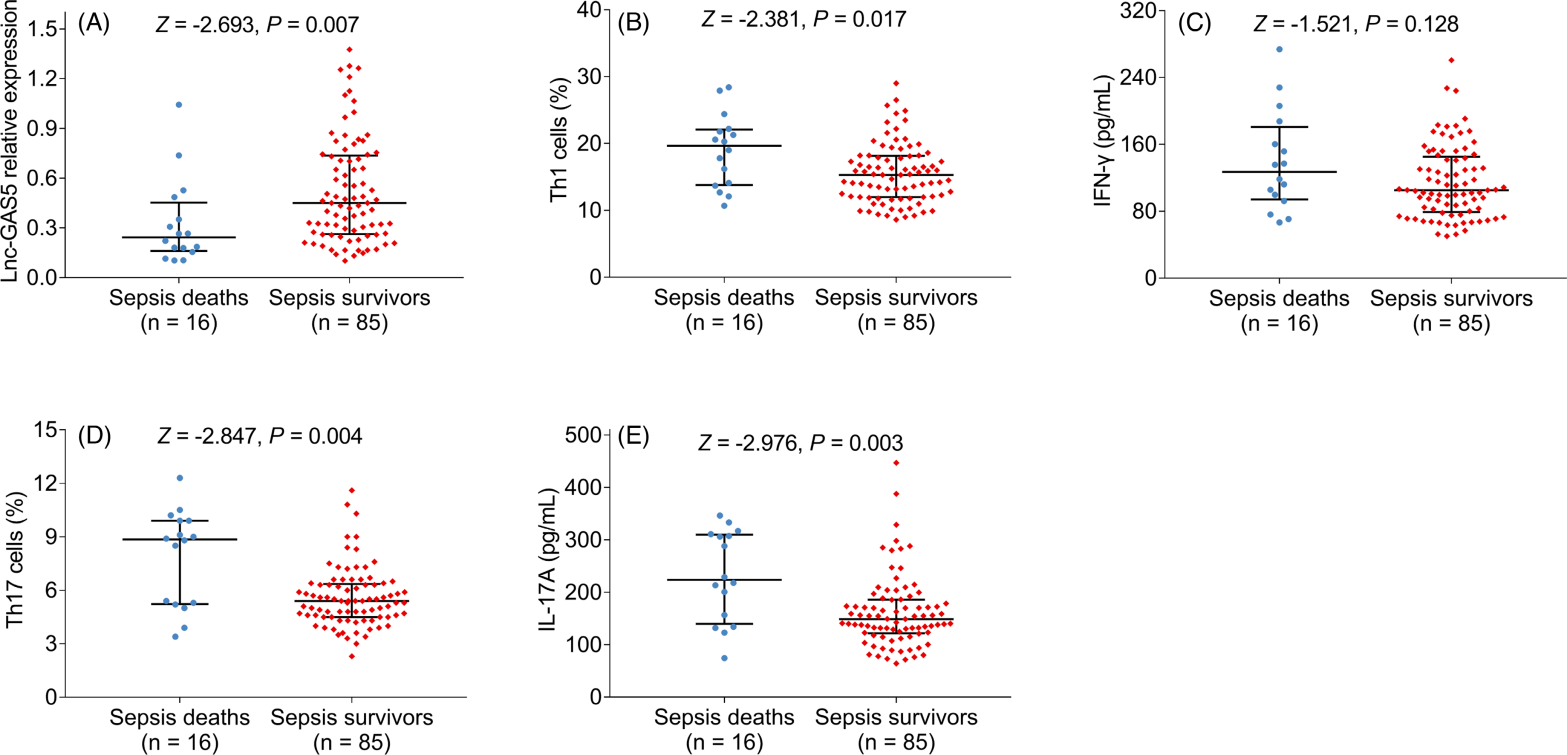
lnc‐GAS5, Th1 cells, IFN‐γ, Th17 cells, and IL‐17A in sepsis deaths and survivors. Comparison of lnc‐GAS5 (A), Th1 cells (B), IFN‐γ (C), Th17 cells (D), and IL‐17A (E) between sepsis deaths and sepsis survivors

**FIGURE 6:**
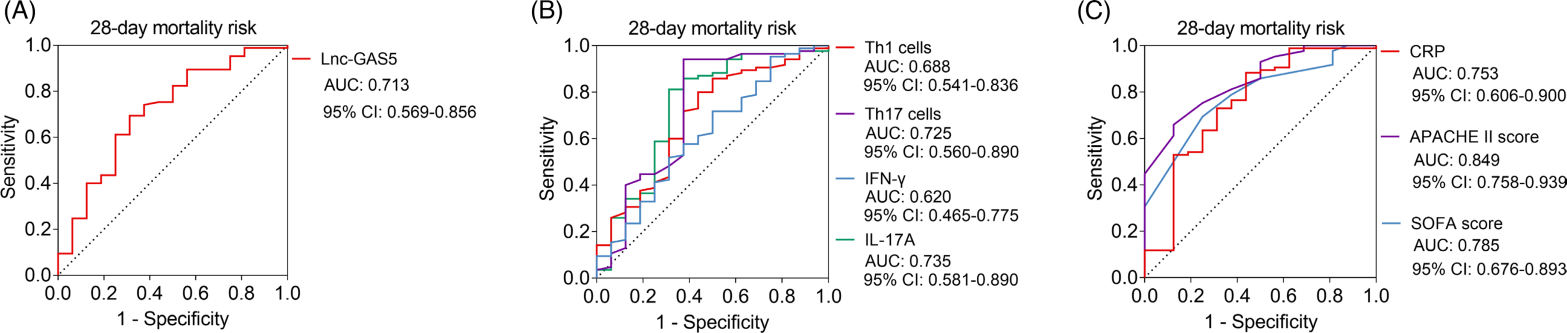
lnc‐GAS5 and other indexes in estimating the 28‐day mortality in sepsis patients. The ability of lnc‐GAS5 (A); Th1 cells, Th17 cells, IFN‐γ, and IL‐17A (B); CRP, APACHE II score, and SOFA score (C) in predicting the sepsis patients with 28‐day mortality

lnc‐GAS5 was decreased in sepsis deaths compared with sepsis survivors (*p* = 0.007), while the Th1 cells (*p* = 0.017), Th17 cells (*p* = 0.004), and IL‐17A (*p* = 0.003) were elevated in sepsis deaths compared with sepsis survivors; the IFN‐γ was of no difference between sepsis deaths and sepsis survivors (*p* = 0.128, Figure 5A–E). Further ROC curve analysis exhibited that lnc‐GAS5 (AUC: 0.713), Th1 cells (AUC: 0.688), Th17 cells (AUC: 0.725), IFN‐γ (AUC: 0.620), IL‐17A (AUC: 0.735), CRP (AUC: 0.753), APACHE II score (AUC: 0.849) and SOFA score (AUC: 0.785) could predict the 28 days mortality risk (Figure 6A–C).

## 4 DISCUSSION

Sepsis is featured by the extremely high mortality which is regarded as a global health priority by the WHO, therefore, it is urgent to find the biomarker involving the development and progression of sepsis to stratify the patients and individualize their treatment, which might help to improve the prognosis of sepsis patients.^1^, ^2^, ^3^, ^4^, ^5^, ^6^ Previous studies have reported the regulation role of lnc‐GAS5 in sepsis.^18^, ^24^ For instance, one study shows lnc‐GAS5‑mediated miR‑23a‑3p promotes inflammation and cell apoptosis by targeting TLR4 in a cell model of sepsis. Another study indicates that lnc‐GAS5 aggravates myocardial depression in mice with sepsis via the microRNA‐449b/HMGB1 axis and the NF‐κB signaling pathway. However, these studies are performed in vivo or in vitro, no study detects the clinical role of lnc‐GAS5 in sepsis patients. Therefore, this study is conducted.

The lnc‐GAS5 has been reported to be involved in regulating macrophage polarization, Treg cell differentiation, and inflammation in various inflammation‐related diseases. For instance, in childhood pneumonia, lnc‐GAS5 might regulate the Treg/Th17 imbalance by targeting the miR‐217/STAT5 axis.^15^ Another study exhibits that in monocytes, knockdown of lnc‐GAS5 might downregulate the macrophage M2 surface markers and concomitant increase in M1 markers, which implies that lnc‐GAS5 could promote the polarization of macrophage to M2 type but inhibit its polarization to M1 type.^21^ T cell differentiation into Th1 cell and Th17 cell plays an important role in the pathogenesis role in sepsis.^25^, ^26^ For instance, one study shows that Th17 cells induced immunosuppression plays a vital role in sepsis.^25^ In our study, we found that lnc‐GAS5 negatively correlated with Th17 cell and its secreted inflammatory cytokine (IL‐17A), also it negatively associated with CRP which might be explained that (1) lnc‐GAS5 inhibited the T cell differentiation into Th17 cells, therefore it negatively associated with Th17 cells and IL‐17A in sepsis, and (2) lnc‐GAS5 restrained the inflammatory response, hence, it negatively correlated with CRP level in sepsis patients.

lnc‐GAS5 serves as a biomarker in indicating the tissue injury in various complex disease, such as the Parkinson's disease, spinal cord injury, sepsis, and ischemic stroke.^17^, ^27^, ^28^, ^29^ While the relationship of lnc‐GAS5 with sepsis‐induced organ injury has not been reported. In this study, we found that lnc‐GAS5 negatively related to SOFA score, SOFA‐Respiratory system score, SOFA‐Coagulation score, SOFA‐Renal system score. The possible reason might be that: (1) lnc‐GAS5 inhibited the hypoxia‐induced pulmonary arterial smooth muscle cell proliferation by regulating KCNK3 expression, therefore lnc‐GAS5 played a protective role in the lung injury model. Hence, lnc‐GAS5 is negatively related to SOFA‐Respiratory system score,^30^ (2) lnc‐GAS5 might inhibit the renal cell pyroptosis in sepsis‐induced renal injury though inhibiting miR‐579‐3p to activate SIRT1/PGC‐1 alpha/Nrf2 signaling pathway, therefore, lnc‐GAS5 negatively associated with SOFA‐Renal system score,^17^ (3) lnc‐GAS5 could inhibit the inflammatory process, which might regulate the coagulation process, therefore, the lnc‐GAS5 was related to the SOFA‐Coagulation score. However, this hypothesis needed further exploration.

The correlation of lnc‐GAS5 with mortality in sepsis patients is also of great concern. However, up to now, there is still no study reports this issue. In the present study, we showed that lnc‐GAS5 was decreased in sepsis deaths compared with sepsis survivors, also, it had an ability in estimating the 28 days mortality in sepsis. These findings might be explained as follows: lnc‐GAS5 was related to the outbreak of inflammation and multi‐organ injury such as lung injury and renal injury, whose injury was associated with higher mortality risk in sepsis patients, therefore, lnc‐GAS5 could predict the 28 days mortality of sepsis patients.^17^, ^30^

Several limitations were nonnegligible in this study. Firstly, the lnc‐GAS5 was only detected after the enrollment of all subjects, while its multi‐time point detection to monitor the progression of sepsis patients was needed and should be conducted in further study. Secondly, a detailed mechanism of lnc‐GAS5 in sepsis was not investigated. Thirdly, even though 101 sepsis were enrolled, the sample size was still small, which may cause insufficient statistical power. Fourthly, the correlation of lnc‐GAS5 with macrophage polarization should be determined in further study. Fifthly, the quantification of lnc‐GAS5 was relative but not absolute value, which was a little far from the clinical utility; therefore, further study with the absolute‐quantification method was needed. Sixthly, the number of sepsis deaths was low, thus the statistical power of data about the comparison of lnc‐GAS5 between septic survivors and septic deaths might be insufficient, which should be validated in a larger‐sample‐size study.

In conclusion, lnc‐GAS5 relates to Th17 cells, and serves as a potential biomarker for sepsis‐induced multi‐organ injury and mortality risk; therefore, helps the clinicians to stratify the sepsis patients and individualize their treatment.

## CONFLICTS OF INTEREST

The authors declare that they have no competing interests.

## Supporting information

Table S1

Table S2

## Data Availability

The data that support the findings of this study are available from the corresponding author upon reasonable request.
