# Supplementary material for: LncRNA GAS5 relates to Th17 cells and serves as a potential biomarker for sepsis inflammation, organ dysfunctions and mortality risk
Source: J Clin Lab Anal. 2022 Mar 24;36(5):e24309. doi: 10.1002/jcla.24309 (PMC9102497; doi:10.1002/jcla.24309)
Supplement: Supplementary file 1 — Table S1 [file JCLA-36-e24309-s001.docx]

**Supplementary table 1.** Factors predicting the sepsis risk by logistic regression analysis.

| Items | *P* value | OR | 95% CI | |
| --- | --- | --- | --- | --- |
|  |  |  | Lower | Upper |
| **Univariate logistic regression analysis** | | | | |
| Lnc-GAS5 | <0.001 | 0.028 | 0.008 | 0.091 |
| Age (years) | 0.191 | 1.022 | 0.989 | 1.057 |
| Gender (male vs. female) | 0.207 | 1.578 | 0.777 | 3.205 |
| BMI (kg/m^2^) | 0.805 | 1.013 | 0.917 | 1.118 |
| Smoke | 0.782 | 0.937 | 0.590 | 1.488 |
| Drink | 0.871 | 0.942 | 0.460 | 1.931 |
| Scr (mg/dL) | <0.001 | 74.580 | 14.012 | 396.950 |
| Albumin (g/L) | <0.001 | 0.850 | 0.807 | 0.896 |
| WBC (10^9^/L) | <0.001 | 1.672 | 1.376 | 2.031 |
| CRP (mg/L) | 0.881 | 6.598E+12 | 0.000 | 2.071E+181 |
| **Multivariate logistic regression analysis** |  |  |  |  |
| Lnc-GAS5 | 0.005 | 0.035 | 0.003 | 0.369 |
| Scr (mg/dL) | 0.758 | 0.707 | 0.078 | 6.426 |
| Albumin (g/L) | 0.009 | 0.837 | 0.733 | 0.957 |
| WBC (10^9^/L) | <0.001 | 1.831 | 1.344 | 2.494 |

OR, odds ratio; CI, confidential interval; lncRNA GAS5, long noncoding RNA growth arrest-specific transcript 5; BMI, body mass index; Scr, serum creatinine; WBC, white blood cell; CRP, C-reactive protein.
