# Supplementary material for: LncRNA GAS5 relates to Th17 cells and serves as a potential biomarker for sepsis inflammation, organ dysfunctions and mortality risk
Source: J Clin Lab Anal. 2022 Mar 24;36(5):e24309. doi: 10.1002/jcla.24309 (PMC9102497; doi:10.1002/jcla.24309)
Supplement: Supplementary file 2 — Table S2 [file JCLA-36-e24309-s002.docx]

**Supplementary table 2.** Correlation of lnc-GAS5 expression with clinical features in sepsis patients.

| Items | Lnc-GAS5, median (IQR) | *Z/χ^2^* value | *P* value |  |
| --- | --- | --- | --- | --- |
| Age (years) |  | -0.225 | 0.822 |  |
| < 60 years | 0.393 (0.218-0.736) |  |  |  |
| ≥ 60 years | 0.419 (0.266-0.656) |  |  |  |
| Gender |  | -0.844 | 0.399 |  |
| Female | 0.354 (0.199-0.674) |  |  |  |
| Male | 0.434 (0.260-0.715) |  |  |  |
| BMI (kg/m^2^) |  | -0.862 | 0.389 |  |
| < 25 kg/m^2^ | 0.386 (0.220-0.659) |  |  |  |
| ≥ 25 kg/m^2^ | 0.443 (0.266-0.747) |  |  |  |
| Smoke status |  | 1.206 | 0.547 |  |
| Never | 0.351 (0.225-0.679) |  |  |  |
| Former | 0.480 (0.266-0.731) |  |  |  |
| Current | 0.490 (0.290-0.730) |  |  |  |
| History of hypertension |  | -0.920 | 0.358 |  |
| No | 0.386 (0.252-0.741) |  |  |  |
| Yes | 0.427 (0.210-0.598) |  |  |  |
| History of hyperlipidemia |  | -0.072 | 0.943 |  |
| No | 0.382 (0.242-0.738) |  |  |  |
| Yes | 0.435 (0.265-0.559) |  |  |  |
| History of diabetes |  | -0.650 | 0.516 |  |
| No | 0.415 (0.248-0.727) |  |  |  |
| Yes | 0.351 (0.226-0.523) |  |  |  |
| History of CKD |  | -0.762 | 0.446 |  |
| No | 0.393 (0.251-0.708) |  |  |  |
| Yes | 0.652 (0.186-0.772) |  |  |  |
| History of cardiovascular and cerebrovascular diseases |  | -0.635 | 0.525 |  |
| No | 0.422 (0.251-0.733) |  |  |  |
| Yes | 0.351 (0.228-0.592) |  |  |  |
| Primary infection site |  | 1.475 | 0.688 |  |
| Abdominal infection | 0.326 (0.206-0.816) |  |  |  |
| Respiratory infection | 0.486 (0.265-0.737) |  |  |  |
| Skin and soft tissue infection | | 0.477 (0.272-0.747) |  |  |
| Other infections | 0.372 (0.252-0.527) |  |  |  |
| Primary organism |  |  |  |  |
| G- |  | -0.694 | 0.488 |  |
| Negative | 0.427 (0.259-0.742) |  |  |  |
| Positive | 0.377 (0.217-0.656) |  |  |  |
| G+ |  | -1.057 | 0.291 |  |
| Negative | 0.382 (0.242-0.653) |  |  |  |
| Positive | 0.470 (0.260-0.807) |  |  |  |
| Fungus |  | -2.160 | 0.031 |  |
| Negative | 0.443 (0.251-0.733) |  |  |  |
| Positive | 0.265 (0.201-0.295) |  |  |  |
| Others |  | -0.149 | 0.882 |  |
| Negative | 0.410 (0.249-0.709) |  |  |  |
| Positive | 0.385 (0.204-0.742) |  |  |  |
| Total culture negative |  | -0.073 | 0.942 |  |
| Negative | 0.415 (0.234-0.725) |  |  |  |
| Positive | 0.400 (0.252-0.683) |  |  |  |

lncRNA GAS5, long noncoding RNA growth arrest-specific transcript 5; IQR, interquartile range; BMI, body mass index; CKD, chronic kidney disease; G-, gram-negative bacillus; G+, gram-positive bacillus.
